# Avian Influenza Virus A(H5N1) Genotype D1.1 Is Better Adapted to Human Nasal and Airway Organoids Than Genotype B3.13

**DOI:** 10.1093/infdis/jiaf598

**Published:** 2025-11-24

**Authors:** Xiaojuan Zhang, Stephanie Joy-Ann Lam, Lin-Lei Chen, Carol Ho-Yan Fong, Wan-Mui Chan, Jonathan Daniel Ip, Shaofeng Deng, Siwen Liu, Rachel Chun-Yee Tam, Pui Wang, Kwok-Hung Chan, King-Pui Florence Chan, James Chung-Man Ho, Jie Zhou, Kwok-Yung Yuen, Honglin Chen, Kelvin Kai-Wang To

**Affiliations:** State Key Laboratory of Emerging Infectious Diseases, Carol Yu Centre for Infection, Department of Microbiology, Li Ka Shing Faculty of Medicine, School of Clinical Medicine, University of Hong Kong, Pokfulam, Hong Kong Special Administrative Region, People's Republic of China; State Key Laboratory of Emerging Infectious Diseases, Carol Yu Centre for Infection, Department of Microbiology, Li Ka Shing Faculty of Medicine, School of Clinical Medicine, University of Hong Kong, Pokfulam, Hong Kong Special Administrative Region, People's Republic of China; Centre for Virology, Vaccinology and Therapeutics, Hong Kong Science and Technology Park, Pak Shek Kok, Hong Kong Special Administrative Region, People’s Republic of China; State Key Laboratory of Emerging Infectious Diseases, Carol Yu Centre for Infection, Department of Microbiology, Li Ka Shing Faculty of Medicine, School of Clinical Medicine, University of Hong Kong, Pokfulam, Hong Kong Special Administrative Region, People's Republic of China; Centre for Virology, Vaccinology and Therapeutics, Hong Kong Science and Technology Park, Pak Shek Kok, Hong Kong Special Administrative Region, People’s Republic of China; State Key Laboratory of Emerging Infectious Diseases, Carol Yu Centre for Infection, Department of Microbiology, Li Ka Shing Faculty of Medicine, School of Clinical Medicine, University of Hong Kong, Pokfulam, Hong Kong Special Administrative Region, People's Republic of China; Centre for Virology, Vaccinology and Therapeutics, Hong Kong Science and Technology Park, Pak Shek Kok, Hong Kong Special Administrative Region, People’s Republic of China; State Key Laboratory of Emerging Infectious Diseases, Carol Yu Centre for Infection, Department of Microbiology, Li Ka Shing Faculty of Medicine, School of Clinical Medicine, University of Hong Kong, Pokfulam, Hong Kong Special Administrative Region, People's Republic of China; State Key Laboratory of Emerging Infectious Diseases, Carol Yu Centre for Infection, Department of Microbiology, Li Ka Shing Faculty of Medicine, School of Clinical Medicine, University of Hong Kong, Pokfulam, Hong Kong Special Administrative Region, People's Republic of China; State Key Laboratory of Emerging Infectious Diseases, Carol Yu Centre for Infection, Department of Microbiology, Li Ka Shing Faculty of Medicine, School of Clinical Medicine, University of Hong Kong, Pokfulam, Hong Kong Special Administrative Region, People's Republic of China; Centre for Virology, Vaccinology and Therapeutics, Hong Kong Science and Technology Park, Pak Shek Kok, Hong Kong Special Administrative Region, People’s Republic of China; State Key Laboratory of Emerging Infectious Diseases, Carol Yu Centre for Infection, Department of Microbiology, Li Ka Shing Faculty of Medicine, School of Clinical Medicine, University of Hong Kong, Pokfulam, Hong Kong Special Administrative Region, People's Republic of China; Centre for Virology, Vaccinology and Therapeutics, Hong Kong Science and Technology Park, Pak Shek Kok, Hong Kong Special Administrative Region, People’s Republic of China; State Key Laboratory of Emerging Infectious Diseases, Carol Yu Centre for Infection, Department of Microbiology, Li Ka Shing Faculty of Medicine, School of Clinical Medicine, University of Hong Kong, Pokfulam, Hong Kong Special Administrative Region, People's Republic of China; Centre for Virology, Vaccinology and Therapeutics, Hong Kong Science and Technology Park, Pak Shek Kok, Hong Kong Special Administrative Region, People’s Republic of China; State Key Laboratory of Emerging Infectious Diseases, Carol Yu Centre for Infection, Department of Microbiology, Li Ka Shing Faculty of Medicine, School of Clinical Medicine, University of Hong Kong, Pokfulam, Hong Kong Special Administrative Region, People's Republic of China; Centre for Virology, Vaccinology and Therapeutics, Hong Kong Science and Technology Park, Pak Shek Kok, Hong Kong Special Administrative Region, People’s Republic of China; State Key Laboratory of Emerging Infectious Diseases, Carol Yu Centre for Infection, Department of Microbiology, Li Ka Shing Faculty of Medicine, School of Clinical Medicine, University of Hong Kong, Pokfulam, Hong Kong Special Administrative Region, People's Republic of China; Centre for Virology, Vaccinology and Therapeutics, Hong Kong Science and Technology Park, Pak Shek Kok, Hong Kong Special Administrative Region, People’s Republic of China; Department of Medicine, School of Clinical Medicine, University of Hong Kong, Pokfulam, Hong Kong Special Administrative Region, People's Republic of China; Department of Medicine, School of Clinical Medicine, University of Hong Kong, Pokfulam, Hong Kong Special Administrative Region, People's Republic of China; State Key Laboratory of Emerging Infectious Diseases, Carol Yu Centre for Infection, Department of Microbiology, Li Ka Shing Faculty of Medicine, School of Clinical Medicine, University of Hong Kong, Pokfulam, Hong Kong Special Administrative Region, People's Republic of China; Centre for Virology, Vaccinology and Therapeutics, Hong Kong Science and Technology Park, Pak Shek Kok, Hong Kong Special Administrative Region, People’s Republic of China; Pandemic Research Alliance Unit, University of Hong Kong, Pokfulam, Hong Kong Special Administrative Region, People's Republic of China; State Key Laboratory of Emerging Infectious Diseases, Carol Yu Centre for Infection, Department of Microbiology, Li Ka Shing Faculty of Medicine, School of Clinical Medicine, University of Hong Kong, Pokfulam, Hong Kong Special Administrative Region, People's Republic of China; Centre for Virology, Vaccinology and Therapeutics, Hong Kong Science and Technology Park, Pak Shek Kok, Hong Kong Special Administrative Region, People’s Republic of China; Pandemic Research Alliance Unit, University of Hong Kong, Pokfulam, Hong Kong Special Administrative Region, People's Republic of China; Department of Microbiology, Queen Mary Hospital, Pokfulam, Hong Kong Special Administrative Region, People's Republic of China; Department of Infectious Disease and Microbiology, The University of Hong Kong–Shenzhen Hospital, Shenzhen, People's Republic of China; State Key Laboratory of Emerging Infectious Diseases, Carol Yu Centre for Infection, Department of Microbiology, Li Ka Shing Faculty of Medicine, School of Clinical Medicine, University of Hong Kong, Pokfulam, Hong Kong Special Administrative Region, People's Republic of China; Centre for Virology, Vaccinology and Therapeutics, Hong Kong Science and Technology Park, Pak Shek Kok, Hong Kong Special Administrative Region, People’s Republic of China; Pandemic Research Alliance Unit, University of Hong Kong, Pokfulam, Hong Kong Special Administrative Region, People's Republic of China; State Key Laboratory of Emerging Infectious Diseases, Carol Yu Centre for Infection, Department of Microbiology, Li Ka Shing Faculty of Medicine, School of Clinical Medicine, University of Hong Kong, Pokfulam, Hong Kong Special Administrative Region, People's Republic of China; Centre for Virology, Vaccinology and Therapeutics, Hong Kong Science and Technology Park, Pak Shek Kok, Hong Kong Special Administrative Region, People’s Republic of China; Pandemic Research Alliance Unit, University of Hong Kong, Pokfulam, Hong Kong Special Administrative Region, People's Republic of China; Department of Microbiology, Queen Mary Hospital, Pokfulam, Hong Kong Special Administrative Region, People's Republic of China; Department of Infectious Disease and Microbiology, The University of Hong Kong–Shenzhen Hospital, Shenzhen, People's Republic of China

**Keywords:** cattle H5N1, clade 2.3.4.4b, genotype D1.1 and B3.13, human respiratory organoid, sialic acid receptor

## Abstract

Three critically ill or fatal avian influenza A(H5N1) human infections have been reported in North America since November 2024. Notably, all were infected with genotype D1.1 instead of B3.13, the dominant genotype before November 2024. Here, we demonstrated that D1.1 could replicate to higher titers in human nasal and airway organoid–derived transwell monolayers from 6 donors. D1.1 exhibited a better binding to α2,3- and α2,6-linked sialic acid than B3.13. No significant differences in most inflammatory or antiviral cytokines/chemokines were observed. These observations suggest that D1.1 is better adapted to both the upper and lower human respiratory tract epithelium than B3.13.

## Teaser

Using human respiratory organoid–derived transwell monolayers, we demonstrated that cattle H5N1 genotype D1.1 replicated more efficiently than B3.13. D1.1 also exhibited a stronger binding to sialic acid receptors than B3.13. Our results demonstrated D1.1's enhanced adaptation to human respiratory epithelium.

The influenza virus A(H5N1) clade 2.3.4.4b was the first avian influenza virus to cause a major outbreak of human infections in North America. The first case of this human H5N1 outbreak occurred in late March 2024, shortly after detection of H5N1 clade 2.3.4.4b viruses in dairy cows and unpasteurized milk samples in the United States [1]. As of 14 May 2025, a total of 72 cases of human H5N1 infections have been reported in the United States, Canada, and Mexico [1–3]. Unlike previous human H5N1 cases, which were associated with a case-fatality rate of over 50%, most clade 2.3.4.4b human cases in North America were mild. However, 2 fatal cases and 1 critically ill case have been reported [2–4].

In an analysis of mild nonhospitalized human H5N1 clade 2.3.4.4b cases between March and October 2024, Garg et al showed that 84.6% (22/26) were infected with the genotype B3.13, while only 15.4% (4/26) patients were infected with the genotype D1.1 [5] (Supplementary Figure 1). In contrast, all 3 fatal or critically ill cases were infected with genotype D1.1 [2–4] (Supplementary Figure 1*B* and Supplementary Table 1). The disproportionately high incidence of D1.1 among fatal or critically ill cases suggests that D1.1 may be more virulent in humans compared to B3.13.

Both B3.13 and D1.1 belong to clade 2.3.4.4b and are reassortants of the Eurasian and American lineages. However, they exhibit significant genomic differences. For B3.13, the hemagglutinin (HA), polymerase acidic (PA), neuraminidase (NA), and matrix (M) gene segments belong to the Eurasian lineage, whereas the polymerase basic 2 (PB2), polymerase basic 1 (PB1), nucleoprotein (NP), and non-structural (NS) segments originate from the North American lineage (https://github.com/USDA-VS/GenoFLU). In contrast, for D1.1, the NA and PA segments are derived from the North American lineage while the PB1 and NS belong to the Eurasian linage. Whether these genomic differences result in differences in human adaptability is currently not known. In this study, we sought to assess the risk of D1.1 to humans in human respiratory organoid–derived transwell monolayers.

## MATERIALS AND METHODS

### Viruses

All H5N1 influenza viruses used in this study were rescued using reverse genetics as we described previously with modifications [6]. The 8 gene segments of the B3.13 A/dairy cow/Texas/008749-003/2024 (Global Initiative on Sharing All Influenza Data [GISAID] number: EPI_ISL_19014386) or the D1.1 A/British Columbia/PHL-2032/2024-11-01 (GISAID number: EPI_ISL_19548836) were cloned into a pHW2000 plasmid system and used as backbones. All viruses were cultured in Madin-Darby canine kidney cell line (ATCC, catalog number CCL-34). The viral genomes were verified using Sanger sequencing. All experiments involving live H5N1 virus were performed in our biosafety level 3 facility.

### Establishment of Human Nasal and Airway Organoids

The collection of clinical specimens for generation of organoids has been approved by the Institutional Review Board of the University of Hong Kong/Hospital Authority of Hong Kong West Cluster (HKU/HA HKW IRB) (reference numbers UW 21-695 and UW 23-376). The human respiratory organoids were derived from 3 female and 3 male donors aged 21–65 years (Supplementary Table 2). Human nasal organoids N-4, N-7, and N-9 were generated from brushings of the midturbinate as described previously [7]. Airway organoid L-12 was generated from resected normal lung tissue as described previously [8]. Airway organoids Aw-1 and Aw-56 were generated from bronchoalveolar lavage fluid (BALF) following the protocol described by Liu et al [9], with minor modifications. In brief, BALF samples were digested using a digestion buffer, centrifuged, and washed. Resultant single cells were embedded in Matrigel and cultured in expansion medium. All organoids were passaged every 7–14 days and maintained at 37°C with 5% CO₂, with medium changes every 2–3 days.

To generate differentiated human nasal organoid–derived transwell monolayers (hNOtm) and human lower airway organoid–derived transwell monolayers (hLAOtm), undifferentiated 3D organoids were dissociated into single cells using 10X TrypLE Select (3–5 minutes, 37°C). Cells were seeded onto Transwell inserts (Corning) and expanded in expansion medium until 90% confluency. Differentiation was induced by replacing the medium with differentiation medium (PneumaCult-ALI Medium, STEMCELL Technologies) for 10–12 days. Medium was added to both apical and basolateral chambers and was replenished every other day.

### Viral Replication in hNOtm and hLAOtm

H5N1 viruses were inoculated to the apical chamber of hNOtm and hLAOtm at a multiplicity of infection (MOI) of 0.01. For negative control, basal medium without virus was added. After incubation at 37°C and 5% CO_2_ for 1 hour, the inoculant was removed, and the apical chambers were washed and replenished with fresh basal medium. The infected organoid cells were incubated at 37°C in a 5% CO_2_ incubator. The supernatants were collected from the apical chambers at 2, 24, 48, and 72 hours postinfection. At each time point, the viral titer in the culture supernatant was determined by plaque assay. Viral replication assay was performed in triplicate in 2 independent experiments.

### Cytokine and Chemokine Assay

The levels of cytokine and chemokine in the culture supernatant were quantified using multiplex panel (LEGENDplex, BioLegend, catalog number 741270) and the manufacturer's protocol was followed. The cytokine and chemokine panel included human granulocyte-macrophage colony-stimulating factor, IFN-α2, IFN-β, IFN-γ, IFN-λ1(IL-29), IFN-λ2 (IL-28A), IL-1β, IL-6, IL-8 (CXCL8), IL-10, IL-12p70, IP-10 (CXCL10), and TNF-α. Data were acquired by BD Bioscience LSRFortessa and analyzed with LEGENDplex data analysis software suite from Qognit.

### Receptor Binding Assay

Receptor binding specificity was analyzed by a solid-phase direct binding assay with 3′-sialyllactose-polyacrylamide-biotin (3′SL, GlycoTech Corporation, catalog number OS165232) and 6′-sialyllactose-polyacrylamide-biotin (6′SL, GlycoTech Corporation, catalog number OS165231). Details of this assay are described in the Supplementary Methods.

### Statistical Analysis

Statistical analysis was performed using GraphPad Prism version 10.4.1 software. Multiple unpaired *t* test was used for the comparison of log-transformed viral titers between B3.13 and D1.1. Paired *t* test was used for the comparison of the levels of cytokines and chemokines. A *P* value of <.05 was considered statistically significant.

## RESULTS

### Prevalence of H5N1 Genotypes Detected in Patients From North America

First, we determined the prevalence of D1.1 in humans from the North America human outbreak. We retrieved all available H5N1 clade 2.3.4.4b sequences from humans in North America from 1 January 2024 to 10 May 2025 shared via GISAID [10]. A total of 53 strains were identified, of which 6 strains had 2 sequences deposited. These 53 strains were collected from patients between 28 March 2024 and 12 February 2025 (Supplementary Table 3). Of these 53 strains, 22 (41.5%) were assigned to genotype B3.13, 8 (15.1%) were assigned to D1.1, 1 (1.9%) was assigned to D1.3, and 22 (41.5%) could not be assigned to any genotypes according to GenoFLU version 1.06 (https://github.com/USDA-VS/GenoFLU).

All strains collected between March and September 2024 belonged to B3.13 (Supplementary Figure 1*C*). D1.1 first emerged in October 2024 and accounted for 32% (8/25) of strains with a successful genotype assigned and collected between September 2024 and February 2025. All strains collected in 2025 belong to genotype D1.

### Comparison of Replication and Cytokines/Chemokines Response Between Genotypes D1.1 and B3.13

**Figure 1.**
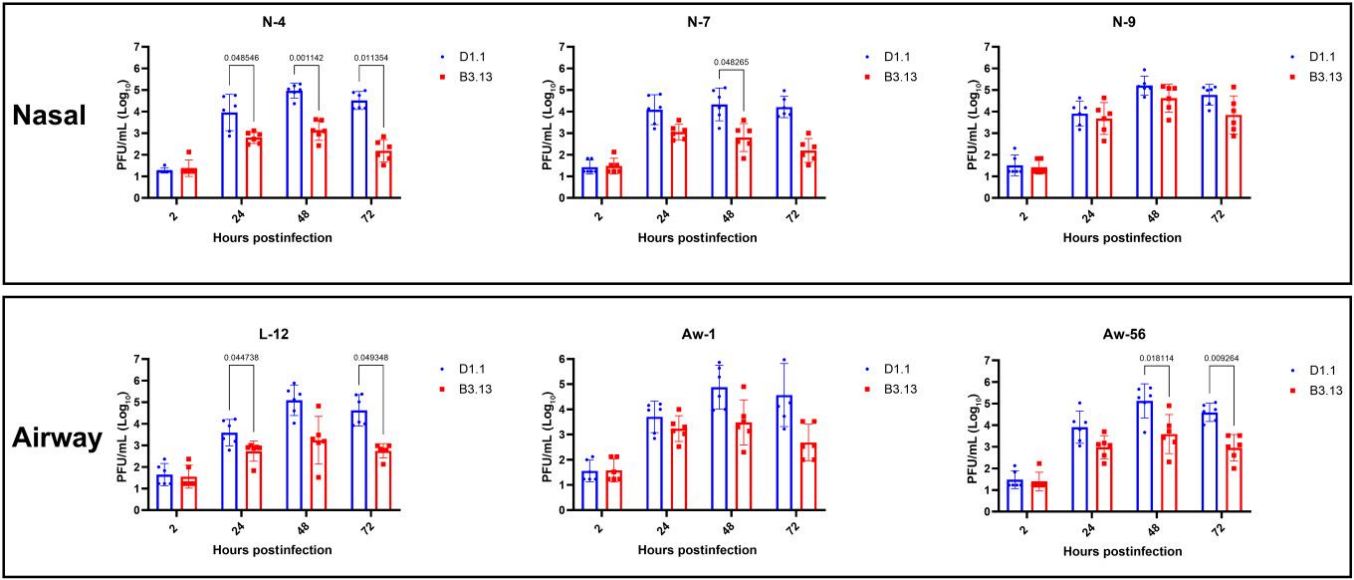
Comparison of viral replication between B3.13 and D1.1 in differentiated human nasal organoid–derived (hNOtm) (N-4, N-7, N-9) and human lower airway organoid–derived transwell monolayers (hLAOtm) (L-12, Aw-1, Aw-56). Differentiated hNOtm and hLAOtm were infected with B3.13 or D1.1 at a multiplicity of infection of 0.01. Each data point represents the viral titer in the culture supernatant of each culture well. In total, there are 6 data points, with 3 data points for each independent experiment. Statistical analysis was performed using multiple unpaired *t* test with log-transformed viral titers. Data represent geometric mean with 95% confidence intervals. Abbreviation: PFU, plaque-forming unit.

To assess whether D1.1 is better adapted to humans than B3.13, we compared the viral replication of D1.1 and B3.13 in hNOtm and hLAOtm. We used reverse genetics–derived H5N1 strains. The recombinant genotype B3.13 A/dairy_cattle/Texas/24-008749-003-recombinant/2024(H5N1) was constructed based on A/dairy_cow/Texas/008749-003/2024 [6, 11, 12], and genotype D1.1 A/British_Columbia/PHL-2032-recombinant/2025 was constructed based on A/British_Columbia/PHL-2032/2024 [2] (Supplementary Table 4). The amino acid differences between the 2 strains are shown in Supplementary Table 5. A total of 6 adult stem cell–derived human respiratory organoids were tested, including 3 hNOtm (N-4, N-7, and N-9) representing the upper respiratory tract, and 3 hLAOtm (L-12, Aw-1, and Aw-56) representing the lower respiratory tract. The peak titers for D1.1 and B3.13 reached 4–5 logs and 3–4 logs, respectively, and were similar between hNOtm and hLAOtm (Figure 1). The viral titers of D1.1 were statistically significantly higher than those of B3.13 in 2 of 3 hNOtm (N-4 and N-7) and 2 of 3 hLAOtm (L-12 and Aw-56) in at least 1 time point. For N-9 and Aw-1, the viral titers were higher for D1.1 than B3.13 at all time points, though not statistically significant.

Severe influenza is associated with marked cytokine activation. Previous studies have shown that B3.13 could cause elevated lung proinflammatory cytokines in mice [11]. We compared the levels of 13 cytokines/chemokines in the culture supernatant of hNOtm and hLAOtm infected with D1.1 and B3.13 at 48 hours postinfection. There were no statistically significant differences for all cytokines/chemokines, except that D1.1 had a slightly higher level of IL-1β than B3.13 in hLAOtm (Supplementary Figure 2).

### Comparison of Sialic Acid Binding Specificities Between Genotypes D1.1 and B3.13

**Figure 2.**
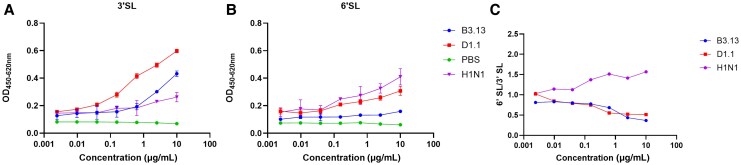
Characterization of the receptor-binding properties of H5N1 virus. Four-fold serial dilutions of 3′-sialyllactose-polyacrylamide-biotin (3′SL) and 6′-sialyllactose-polyacrylamide-biotin (6′SL) coating microtiter plates were incubated with 32 hemagglutinin units of the indicated viruses or phosphate-buffered saline (PBS; negative control). After washing, virus binding was detected by an anti–influenza A nucleoprotein antibody and a horseradish peroxidase–conjugated secondary antibody. The optical density (OD) values for each condition with each virus or PBS are shown. The experiment was performed in triplicate. Data represent mean ± standard deviation. *A*, Binding to 3′SL. *B*, Binding to 6′SL. *C*, 6′SL/3′SL ratio.

To understand why D1.1 replicated better in hNOtm and hLAOtm than B3.13, we compared the receptor binding preference between the 2 viruses. D1.1 showed stronger binding to 3′SL and 6′SL than B3.13 (Figure 2*A* and 2*B*), but there was no difference in 6′SL/3′SL ratio between these 2 viruses (Figure 2*C*).

## DISCUSSION

All reported fatal or critically ill H5N1 cases up to April 2025 in North America have been infected with the genotype D1.1 [2–4]. Here, we demonstrated that D1.1 replicated more robustly in human organoid–derived transwell monolayers from either the upper or lower respiratory tract of 6 donors, although no differences in cytokines/chemokines response were observed. We also found that D1.1 had better binding to 6′SL and 3′SL than B3.13.

Gu et al previously showed that at 37°C, a genotype B3.13 strain isolated from a patient (HuTX-37) replicated to a similar level as B3.13 isolated from milk of a lactating dairy cow (NM93) in primary human alveolar cells [13], although the replication of HuTX-37 was more robust than NM93 at 33°C. Flagg et al reported that a patient's B3.13 H5N1 strain (A/Texas/37/2024) replicated more efficiently than a dairy cow B3.13 H5N1 strain (A/bovine/Ohio/B24OSU-342/2024) at 37°C in 2 human lung organoids, though not statistically significant [14]. In contrast, we demonstrated that a reverse genetically constructed D1.1 strain replicated better than a B3.13 strain in respiratory organoids derived from 6 donors even at 37°C, the temperature of the lower human respiratory tract. Hence, the more severe disease associated with D1.1 could be related to the more robust replication in the human lower respiratory tract.

Flagg et al reported that a dairy cow strain of B3.13 could elicit much higher levels of IFN-β, IL-6, and IL-1β than a human B3.13 strain in their adult stem cell–derived human lung organoid [14]. In contrast, we did not observe any significant differences for most cytokines/chemokines from respiratory organoids between D1.1 and B3.13. Therefore, the difference in clinical severity between D1.1 and B3.13 could not be explained by the inflammatory response from airway epithelial cells. One limitation in our organoid model is the lack of immune cells. Resident innate immune cells in the respiratory tract such as alveolar macrophages and dendritic cells play a significant role in inducing inflammatory and antiviral cytokine/chemokine response. Further studies comparing the response from these resident innate immune cells would be warranted.

The reverse genetically derived D1.1 strain we used in this experiment was constructed based on the D1.1 from a critically ill patient and contained 3 markers of human adaptation, including HA E186D, HA Q222H, and PB2 E627K [2]. HA Q222L and PB2 E627K have been associated with airborne transmission of H5N1 virus [15]. Further studies are required to delineate whether the difference in replication between D1.1 and B3.13 is related to these markers.

Our observations suggest that D1.1 genotype may be better adapted to humans than B3.13. Further studies are required to determine if there are differences in the replication of different strains within the same clade. As D1.1 is now widespread among dairy cows in the United States, there is an increasing risk of further adaptation of D1.1 with higher transmissibility or virulence among mammals. Continuous phenotypic monitoring using human organoids and other in vivo models will provide critical information for assessing the risk of D1.1 or other novel genotypes in humans.

## Supplementary Material

jiaf598_Supplementary_Data

## Supplementary Data

Supplementary materials are available at *The Journal of Infectious Diseases* online (http://jid.oxfordjournals.org/). Supplementary materials consist of data provided by the author that are published to benefit the reader. The posted materials are not copyedited. The contents of all supplementary data are the sole responsibility of the authors. Questions or messages regarding errors should be addressed to the author.

## Notes

***Acknowledgments.*** We gratefully acknowledge the authors originating and submitting laboratories of the influenza H5N1 genetic sequences and metadata made available through GISAID on which this research is based (Supplementary Table 3).

***Author contributions.*** X. Z., S. J.-A. L., and L.-L.C. had roles in study design, data collection, data analysis, data interpretation, and writing of the manuscript. K. K.-W. T. had roles in study design, data analysis, data interpretation, and writing of the manuscript. C. H.-Y. F., W.-M. C., J. D. I., S. D., S. L., R. C.-Y. T., P. W., and K.-H. C. had roles in performing the experiments, data collection, data analysis, and/or data interpretation. K.-P. F. C. and J. C.-M. H. had roles in patient recruitment. J. Z., K.-Y. Y., and H. C. had roles in study design and data interpretation. All authors revised the manuscript critically for important intellectual content and approved the manuscript.

***Financial support.*** This study was partly supported by the Partnership Programme of Enhancing Laboratory Surveillance and Investigation of Emerging Infectious Diseases and Antimicrobial Resistance for the Department of Health, the Hong Kong Special Administrative Region government; and the InnoHK initiative of the Innovation and Technology Commission of the Hong Kong Special Administrative Region government. We acknowledge funding received from private donors including Richard Yu and Carol Yu, Shaw Foundation Hong Kong, Michael Seak-Kan Tong, May Tam Mak Mei Yin, Lee Wan Keung Charity Foundation Limited, Providence Foundation Limited (in memory of the late Lui Hac-Minh), Hong Kong Sanatorium & Hospital, Respiratory Viral Research Foundation Limited, Hui Ming, Hui Hoy and Chow Sin Lan Charity Fund Limited, Chan Yin Chuen Memorial Charitable Foundation, and Marina Man-Wai Lee.
