# Supplementary material for: Avian Influenza Virus A(H5N1) Genotype D1.1 Is Better Adapted to Human Nasal and Airway Organoids Than Genotype B3.13
Source: J Infect Dis. 2025 Nov 24;233(3):e662–6. doi: 10.1093/infdis/jiaf598 (PMC13017197; doi:10.1093/infdis/jiaf598)
Supplement: jiaf598_Supplementary_Data [file jiaf598_supplementary_data.zip › 4_Supplementary_Figures_Table_20251025.docx]

**Supplementary Materials**

**Supplementary Figure 1.** Prevalence of H5N1 clade 2.3.4.4b genotypes among human cases in North America between March 2024 and February 2025. (A, B) Proportion of genotypes among (A) mild and (B) severe cases. The genotype information for mild cases was retrieved from the publication of Garg S et al [1]. The genotype information of severe cases was retrieved from references listed in Supplementary Table 1. (C) Change in prevalence of genotypes. The genotype information was obtained from GISAID on May 10, 2025 (Supplementary Table 3).


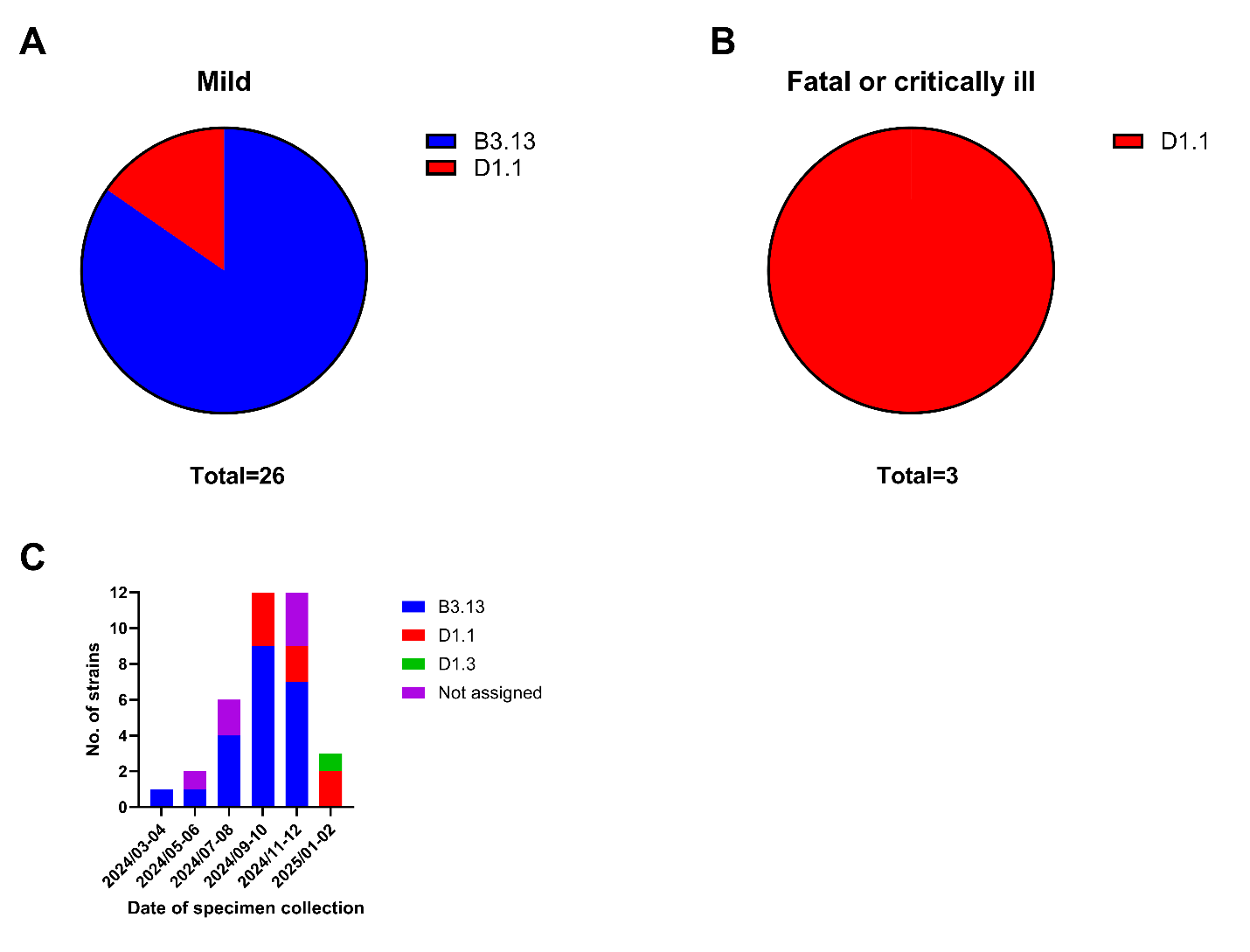


**Supplementary Figure 2.** Cytokine and chemokine response in (A) nasal organoids and (B) airway organoids. The levels of cytokines and chemokines in culture supernatant at 48 hours post infection were measured using the LEGENDplex multiplex panel. Each data point represents the mean of triplicate in each organoid. Horizontal lines represent mean ± standard error of mean. The comparison of the cytokines/chemokines between B3.13 and D1.1 was performed using paired t-test.

**A**

**B**

**Supplementary Table 1**. Hospitalized H5N1 cases reported

| **Date of hospitalization** | **Age**  **(years)** | **Location** | **Outcome** | **Genotype** | **Reference** |
| --- | --- | --- | --- | --- | --- |
| September 2024 | Not reported | Missouri, United States | Discharge from hospital | Not reported | [2] |
| November 2024 | 13 | British Columbia, Canada | Admitted to intensive care unit and required venovenous extracorporeal membrane oxygenation | D1.1 | [3] |
| December 2024 | Not reported | Louisiana, United States | Died | D1.1 | [4] |
| February 2025 | Not reported | Ohio, United States | Discharge from hospital | D1.3 | [5] |
| February 2025 | Not reported | Wyoming, United States | Discharge from hospital | D1.1 | [5] |
| March 2025 | <10 | Durango, Mexico | Died | D1.1 | [6] |

**Supplementary Table 2**. Details of the human respiratory tract organoids used in this study

| **Organoid type** | **Organoid ID** | **Source** | **Age (years)** | **Sex** |
| --- | --- | --- | --- | --- |
| **Nasal** | N-4 | Nasal brushing | 28 | Male |
|  | N-7 | Nasal brushing | 41 | Female |
|  | N-9 | Nasal brushing | 21 | Female |
| **Airway** | L-12 | Normal lung tissue | 65 | Female |
|  | Aw-1 | BAL fluid | 58 | Male |
|  | Aw-56 | BAL fluid | 57 | Male |

Abbreviation: BAL, bronchoalveolar lavage

**Supplementary Table 4.** Reverse genetically derived H5N1 virus used in this study

| **Genotype** | **Recombinant virus** | | **Original reference virus** | |
| --- | --- | --- | --- | --- |
|  | **Isolate name** | **NCBI GenBank Accession number** | **Isolate name** | **GISAID Accession number** |
| **B3.13** | A/dairy_cattle/Texas/24-008749-003-recombinant/2024(H5N1) | PQ898029–PQ898036 | A/dairy_cow/Texas/008749-003/2024 | EPI_ISL_19014386 |
| **D1.1** | A/British_Columbia/PHL-2032-recombinant/2025 | PV612029-PV612036 | A/British_Columbia/PHL-2032/2024 | EPI_ISL_19548836 |

**Supplementary Table 5.** Differences in amino acids between genotype B3.13 strain A/dairy_cattle/Texas/24-008749-003-recombinant/2024(H5N1) and genotype D1.1 strain A/British_Columbia/PHL-2032-recombinant/2025

| **Protein** | **Amino acid residue** | **B3.13**  **(A/dairy cow/Texas/24-008749-003/2024)** | **D1.1**  **(A/British Columbia/PHL-2032/2024-11-01)** |
| --- | --- | --- | --- |
| HA (H5 numbering) | 36 | T | A |
|  | 104 | M | L |
|  | 115 | Q | L |
|  | 140 | A | T |
|  | 186 | E | D |
|  | 194 | I | T |
|  | 210 | A | V |
|  | 222 | Q | H |
|  | 325 | K | R |
|  | 475 | N | D |
|  | 510 | V | I |
| M1 | 82 | S | N |
|  | 85 | S | N |
|  | 87 | T | N |
|  | 200 | V | A |
|  | 227 | T | A |
| M2 | 61 | G | R |
|  | 88 | N | D |
| NA | 8 | T | I |
|  | 16 | V | A |
|  | 19 | I | T |
|  | 20 | V | T |
|  | 23 | M | V |
|  | 44 | Y | N |
|  | 45 | Q | H |
|  | 48 | P | T |
|  | 53 | I | V |
|  | 71 | S | N |
|  | 74 | F | L |
|  | 75 | L | I |
|  | 81 | T | D |
|  | 82 | S | P |
|  | 84 | T | A |
|  | 221 | N | S |
|  | 234 | V | I |
|  | 241 | V | I |
|  | 257 | K | R |
|  | 269 | M | L |
|  | 272 | P | S |
|  | 286 | G | S |
|  | 287 | D | E |
|  | 288 | I | V |
|  | 321 | I | V |
|  | 329 | N | S |
|  | 336 | S | G |
|  | 338 | M | V |
|  | 339 | P | S |
|  | 395 | E | A |
| NP | 52 | H | Y |
|  | 482 | N | S |
| PA | 85 | A | T |
|  | 113 | R | K |
|  | 219 | I | L |
|  | 222 | N | D |
|  | 269 | R | K |
|  | 277 | P | S |
|  | 322 | I | V |
|  | 323 | V | I |
|  | 348 | I | L |
|  | 388 | S | G |
|  | 391 | R | K |
|  | 400 | S | P |
|  | 421 | S | I |
|  | 441 | V | M |
|  | 497 | R | K |
|  | 545 | I | V |
|  | 558 | L | S |
|  | 608 | S | T |
|  | 626 | K | R |
| PA-X | 61 | M | I |
|  | 85 | A | T |
|  | 113 | R | K |
|  | 193 | N | S |
|  | 195 | R | K |
|  | 219 | F | S |
|  | 250 | Q | P |
|  | 252 | R | K |
| PB1-F1 | 4 | E | G |
|  | 7 | I | T |
|  | 8 | P | Q |
|  | 12 | L | S |
|  | 18 | I | T |
|  | 20 | K | R |
|  | 21 | K | R |
|  | 22 | G | E |
|  | 31 | G | E |
|  | 36 | I | T |
|  | 40 | D | G |
|  | 42 | C | Y |
|  | 44 | M | R |
|  | 46 | M | T |
|  | 47 | S | N |
|  | 49 | V | A |
|  | 54 | R | Q |
|  | 55 | T | I |
|  | 57 | S | C |
|  | 58 | L | W |
|  | 65 | K | R |
|  | 66 | N | S |
|  | 68 | I | T |
|  | 70 | E | G |
|  | 75 | R | L |
|  | 76 | V | A |
|  | 82 | L | S |
|  | 84 | N | S |
|  | 90 | S | N |
| PB2 | 58 | A | T |
|  | 109 | I | V |
|  | 139 | I | V |
|  | 255 | A | V |
|  | 362 | G | E |
|  | 382 | I | V |
|  | 441 | N | D |
|  | 495 | I | V |
|  | 627 | E | K |
|  | 631 | L | M |
|  | 649 | I | V |
|  | 676 | A | T |
| NEP | 7 | L | S |
|  | 36 | E | K |
|  | 63 | G | E |
|  | 85 | H | N |
| NS1 | 7 | L | S |
|  | 75 | E | G |
|  | 83 | S | P |
|  | 87 | P | S |
|  | 116 | S | C |
|  | 139 | N | D |
|  | 147 | L | I |
|  | 171 | D | N |
|  | 193 | R | Q |
|  | 223 | E | A |

**REFERENCES**

1. Garg S, Reinhart K, Couture A, et al. Highly Pathogenic Avian Influenza A(H5N1) Virus Infections in Humans. N Engl J Med **2024**; 10.1056/NEJMoa2414610.

2. Centers for Disease Control and Prevention. CDC Confirms Human H5 Bird Flu Case in Missouri. Available at <https://www.cdc.gov/media/releases/2024/s0906-birdflu-case-missouri.html>. Accessed on May 8, 2025. **2025**.

3. Jassem AN, Roberts A, Tyson J, et al. Critical Illness in an Adolescent with Influenza A(H5N1) Virus Infection. N Engl J Med **2024**; 10.1056/NEJMc2415890.

4. Centers for Disease Control and Prevention. Genetic Sequences of Highly Pathogenic Avian Influenza A(H5N1) Viruses Identified in a Person in Louisiana. Available at <https://www.cdc.gov/bird-flu/spotlights/h5n1-response-12232024.html>. Accessed on May 6, 2025. **2025**.

5. Centers for Disease Control and Prevention. Weekly US Influenza Surveillance Report: Key Updates for Week 7, ending February 15, 2025. Available at <https://www.cdc.gov/fluview/surveillance/2025-week-07.html>. Accessed on May 8, 2025. **2025**.

6. World Health Organization. Avian Influenza A(H5N1) - Mexico. Available at <https://www.who.int/emergencies/disease-outbreak-news/item/2025-DON564>. Accessed on May 6, 2025. **2025**.
