# Supplementary material for: Avian Influenza Virus A(H5N1) Genotype D1.1 Is Better Adapted to Human Nasal and Airway Organoids Than Genotype B3.13
Source: J Infect Dis. 2025 Nov 24;233(3):e662–6. doi: 10.1093/infdis/jiaf598 (PMC13017197; doi:10.1093/infdis/jiaf598)
Supplement: jiaf598_Supplementary_Data [file jiaf598_supplementary_data.zip › 5_Supplementary_Methods_JID_20251025.docx]

**SUPPLEMENTARY METHODS**

**Receptor binding assay**

Receptor binding specificity was analyzed by a solid-phase direct binding assay with 3'-sialyllactose-polyacrylamide-biotin (3’SL, GlycoTech Corporation, Catalog no. OS165232) and 6'-sialyllactose-polyacrylamide-biotin (6’SL, GlycoTech Corporation Catalog no. OS165231) as described previously with modifications [1]. Briefly, 4-fold serial dilutions of 3’SL and 6’ SL were prepared in 0.05 M NaHCO_3_ (pH 9.6), and 100 μl of the diluted SA was added to each well of the 96-well microtiter plates (Thermo Fisher Scientific, Catalog no. 442404) and placed in 4 °C overnight. The next day, glycopolymer solutions were removed and the plates were blocked by blocking reagent at room temperature (RT) for 1h. After washing with cold PBS, the plates were incubated in a solution containing 32 haemagglutination (HA) units of H5N1 (B3.13 or D1.1) or H1N1 in PBS at 4°C overnight. After washing with PBS containing 0.1% Tween 20 (PBST), an anti-FluA nucleoprotein antibody [2] were added to each well and the plates were incubated at RT for 1h. The wells were washed with PBST then incubated with horseradish-peroxidase (HRP)-conjugated goat anti-mouse IgG antibodies (Thermo Fisher Scientific, Catalog no. 31430) RT for 1h. The plates were washed again, the reaction was developed by adding 3,3’,5,5’-tetramethylbenzidine single solution (TMB) (Invitrogen, Catalog no. 002023) and stopped with 0.3 N H_2_SO_4_. The optical density (OD) was read at 450 and 620 nm.

**References**

1. Eisfeld AJ, Biswas A, Guan L, et al. Pathogenicity and transmissibility of bovine H5N1 influenza virus. Nature **2024**; 633:426–32.

2. Chen LL, Wu WL, Chan WM, et al. Assessment of population susceptibility to upcoming seasonal influenza epidemic strain using interepidemic emerging influenza virus strains. Epidemiol Infect **2019**; 147:e279.
